# Successful Bosutinib Experience in an Elderly Acute Lymphoblastic Leukemia Patient with Suspected Central Nervous System Involvement Transformed from Chronic Myeloid Leukemia

**DOI:** 10.1155/2015/689423

**Published:** 2015-12-01

**Authors:** Erden Atilla, Pinar Ataca, Elif Ozyurek, Ilhan Erden, Gunhan Gurman

**Affiliations:** ^1^Department of Hematology, Ankara University School of Medicine, 06590 Ankara, Turkey; ^2^Department of Radiology, Ankara University School of Medicine, 06590 Ankara, Turkey

## Abstract

Managing the blast phase in chronic myeloid leukemia (CML) is challenging because limited data are available for elderly patients. The involvement of the central nervous system (CNS) increases the risk of a poor prognosis. Here, we present an elderly blast phase CML patient with suspected CNS involvement who was successfully treated with bosutinib.

## 1. Introduction

Tyrosine kinase inhibitors (TKIs) have created a new paradigm in chronic myeloid leukemia (CML) treatment. In brief, TKIs inhibit BCR-ABL oncoproteins in patients with Philadelphia chromosome positive (Ph+) CML and acute lymphoblastic leukemia (ALL) [1]. The Ph+ chromosome is detected in 95% of CML cases, whereas it is detected in 5–49% of ALL cases [2].

Bosutinib (SKI-606), a dual SRC and ABL inhibitor, differs from imatinib because it minimally inhibits KIT and platelet-derived growth factor receptor (PDGFR) [3]. The efficacy of bosutinib has been shown in phases 1 and 2 trials for all CML stages after treatments with only imatinib or with imatinib plus either nilotinib or dasatinib [4]. In the phase 3 BELA (Bosutinib Efficacy and Safety in Newly Diagnosed CML) trial, bosutinib patients could not reach the primary end point, which was to demonstrate superiority over imatinib in achieving complete cytogenetic responses by the 12th month of therapy. Bosutinib achieved cytogenetic and molecular responses more quickly than imatinib did; however, the survival rates were not significantly different. Grades 3-4 myelosuppression was reported when bosutinib was utilized as a first-line treatment (thrombocytopenia 14%, neutropenia 11%, and anemia 6%) [5].

Treating the lymphoid blast phase, which includes 20–30% of all blast phase CML cases, has been challenging ever since vincristine-prednisolone based therapy was first developed in 1970 [6]. The blast phase progression incidence in CML cases decreased from 20% to 1–1.5% after TKI therapy was introduced [7]. Central nervous system (CNS) involvement is frequently observed in Ph+ ALL and CML blastic crisis cases [8]. In CML, various chromosomal abnormalities can be detected in 2–17% of Ph-cells [9].

Treating older patients with ALL is still a dilemma; so far, very limited data are available. With the use of TKIs upfront in older patients in Ph+ALL, the survival and relapse-free survival were significantly improved (66% versus 43% and 58% versus 11% at 1 year, resp.) [10]. The mortality of induction chemotherapy varies widely (0% to 42%) due to infections and toxicities [11]. In two studies, 30% to 43% of patients older than age 60, compared to 18% to 22% of younger patients, had a performance status of 2 or more [12]. For general management, it is essential to identify fit and unfit patients in treatment decisions [11].

Herein, we present a patient diagnosed with chronic-phase CML that progressed to the blast phase after treatment with imatinib, dasatinib, and nilotinib. The blast phase CML was treated successfully with bosutinib.

## 2. Case

A 72-year-old male patient presented, in 2006, with leukocytosis and hepatosplenomegaly. A complete blood cell analysis showed that the white blood cell count was 114 × 10^9^/L, the hemoglobin was 10.4 g/dL, and the platelet count was 154 × 10^9^/L. A peripheral blood smear showed populations of 33% neutrophils, 6% myeloblasts, 6% promyelocytes, 4% myelocytes, 15% metamyelocytes, 6% lymphocytes, 6% monocytes, 5% basophils, and 4% eosinophils. A bone marrow analysis showed hypercellularity with significant myeloid hyperplasia and 3% myeloblasts. There were no additional chromosomal abnormalities other than t(9;22)(q34;q11). The patient was diagnosed with chronic phase CML with a high Sokal score (1.21). He was initially treated with 400 mg imatinib/day; however, the dose was increased to 800 mg/day after 12 months because a cytogenetic analysis showed Ph+>35%. After 2 years of good drug compliance (in 2008), a major molecular response (MMR) was not achieved; therefore, 100 mg dasatinib/day was started. The patient achieved a complete cytogenetic response (CCyR) in the 6th month and MMR in the 12th month. The dasatinib dose was reduced to 70 mg/day and then 50 mg/day after a grade 3 thrombocytopenia was detected. The patient's treatment was changed to 400 mg nilotinib/twice a day because he lost MMR and complete cytogenetic response two years after nilotinib initiation. A mutation analysis of the BCR-ABL1 kinase domain was negative. He achieved CCyR by the sixth month and MMR by the twelfth month with nilotinib.

**Figure 1:**
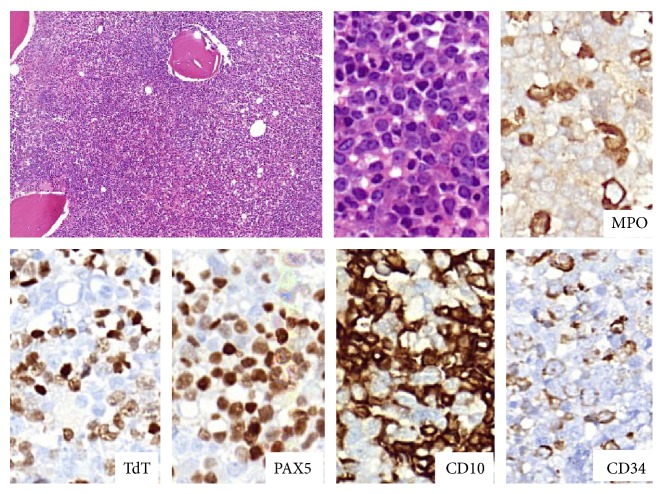
The bone marrow was diffusely infiltrated with blasts. Blasts strongly expressed TdT, Pax5, and CD10. CD34 was partially and weakly expressed by blasts. MPO was negative on blastic cells.

**Figure 2:**
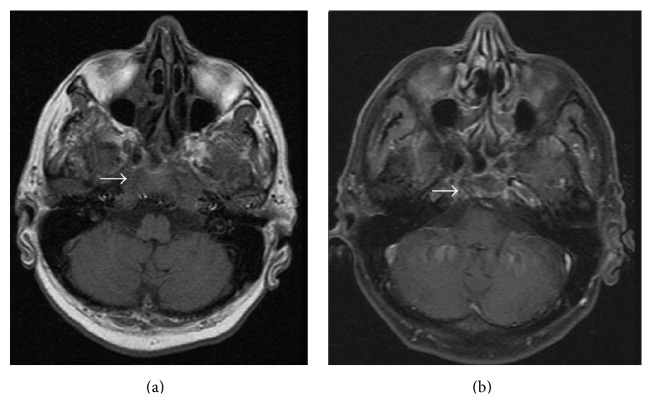
Initial axial enhanced T1-weighted MRI (a) show enhancement (arrow) of the clivus and occipital condyles corresponding to infiltration. Eight months later after systemic therapy axial enhanced T1-weighted MRI (b) shows significant reduction of the enhancement (arrow).

**Figure 3:**
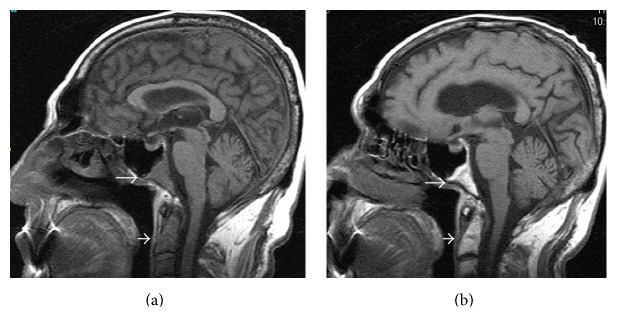
Initial sagittal nonenhanced T1-weighted MRI (a) show hypointensity of the clivus (arrow) and cervical vertebrae (short arrow) corresponding to infiltration. Eight months later after systemic therapy sagittal nonenhanced T1-weighted MRI (b) shows significant reduction of the hypointensity of the both clivus (arrow) and cervical vertebrae (short arrow).

**Figure 4:**
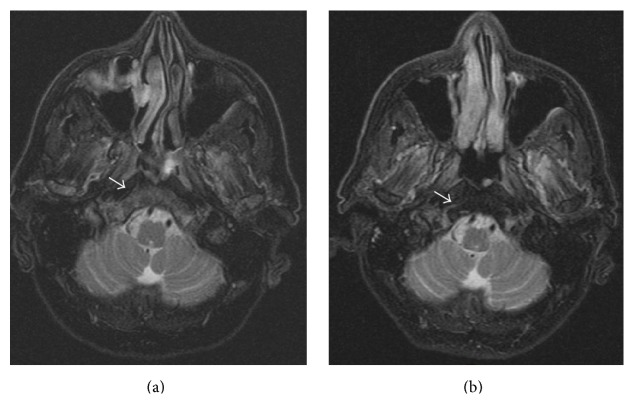
Axial T2 weighted fat-suppressed images before (a) and after therapy (b) clearly show reduction of the pathological hyperintensity of the clivus and occipital condyles corresponding to infiltration.

In December 2013, the patient presented with fatigue and weight loss. His hemogram results were as follows: 56.5 × 10^9^ WBCs/L, 14.6 g Hb/dL, and 71 × 10^9^ platelets/L. The bone marrow biopsy was consistent with 42% blasts diffusely positive with TdT, PAX5, and CD10 and partially positive with CD34 addressing B cell lymphoblastic leukemia (B-ALL) infiltration (Figure 1). The karyotype was 45,XY,-7,t(9;22)(q34;q11.2) at 19/19 metaphases in the bone marrow culture. His performance status was poor. Because he had progressed under imatinib, dasatinib, and nilotinib, we decided to initiate bosutinib treatment. Pretreatment with 10 mg prednisone/m^2^ for 7 days was started, and 40 mg prednisone/m^2^/day by mouth was continued from day 1 to day 45. 500 mg/day of bosutinib was initiated from day 1. CNS prophylaxis was performed six times, with 15 mg methotrexate, 40 mg ARA-C, and 4 mg dexamethasone administered twice weekly. On the 30th day, the patient was hospitalized with a diagnosis of pneumonia and pancytopenia. A control bone marrow biopsy showed that the ALL was in remission. Myelosuppression-related pancytopenia was developed as a side effect of bosutinib. The treatment was temporarily stopped for two weeks and later resumed with a reduced bosutinib dose of 500 mg every other day. Meanwhile, the patient developed depression symptoms and a lack of appetite starting at day 40. A cranial MRG revealed cerebral and cerebellar atrophy as well as contrast on the clivus (CNS involvement) (Figures 2(a), 3(a), and 4(a)). Flow cytometric and cytogenetic analysis of the CSF revealed it to be normal. Radiotherapy was administered at a dose of 200 cGy/day for 10 days and methotrexate and ARA-C intrathecal chemotherapy were administrated six times, twice weekly, for suspected CNS involvement. The clivus involvement disappeared during the follow-up period (Figures 2(b), 3(b), and 4(b)). The patient could not tolerate the methotrexate/vincristine maintenance therapy. We continued with 500 mg bosutinib every other day and followed up at the 3rd, 6th, and 12th months. At the 14th month follow-up, he had a complete hematological response, and his bone marrow was still in remission.

## 3. Discussion

Before the era of TKIs, the American Society of Hematology (ASH) recommended managing CML with conventional chemotherapy, IFN-alpha, and allogeneic stem cell transplantation in 1998 [13]. The European Leukemia Network (ELN) proposed 400 mg imatinib/day as a first-line treatment in their 2006 guidelines [14]. We initiated treatment as recommended and increased the dose to achieve a target response. In the 2009 ELN guidelines, a second-line treatment was suggested with second-line TKIs (nilotinib or dasatinib). Additionally, allogeneic stem cell transplantation was recommended for refractory/blastic or accelerated-phase patients [15]. We switched the treatment first to dasatinib and then to nilotinib for our patient.

Five years after diagnosis, our patient's CML transformed to B cell ALL. In blastic transformation pathogenesis, the cells escaping from tyrosine kinase inhibition gain a growth advantage, which is the major issue that has been demonstrated for this disease [16]. Trisomy 8, isochromosome i(17q), trisomy 19, trisomy 21, trisomy 17, and deletion 7 are cytogenetic anomalies that have been reported to be responsible for blastic phase transformations [17, 18]. Trisomy 8, trisomy 10, and i(17q) have been detected in myeloid blastic crisis cases, which is similar to our case, as monosomy 7 is common during lymphoid blastic crisis [17]. Under imatinib therapy, Kovitz et al. reported secondary myelodysplastic syndrome, acute myeloid leukemia, and ALL in 17 patients; however, monosomy 7 was the most common chromosomal anomaly reported [9]. In 21 of 272 patients (8%) that underwent imatinib treatment, some chromosomal anomalies developed, including trisomy 8, monosomy 5 or 7, and 20q- [19].

Blastic phase treatment is still challenging, and the median survival after a blastic phase diagnosis ranges from 7 to 11 months. Blastic crisis can be managed by decreasing the tumor burden through BCR-ABL elimination with TKIs [16]. Vignetti et al. reported that, upon using the GIMEMA LAL0201-B protocol, imatinib and steroid induction therapy in older ALL patients improved survival up to 20 months with minimal toxicity. The remission rate, survival rate, and disease-free survival rate were reported as 100%, 74%, and 48% after 1 year [20]. Because our patient had a poor performance status, and the toxicity was expected to be high in more intensive induction therapy, we modified the protocol with bosutinib because of the imatinib, dasatinib, and nilotinib refractoriness in our patient. Bosutinib has a comparable effect with nilotinib and dasatinib in CML [21]. In a 134-patient series (63 accelerated phase, 48 blastic phase, and 23 ALL), complete hematologic responses were achieved in 61% of the tumors that were in the accelerated phase and 3% of those that were in the blastic phase, whereas complete cytogenetic responses were detected in 33% of the accelerated phase and 29% of the blastic phase cases with bosutinib. These responses were maintained in 67% of the accelerated phase cases over one year; however, the responses were not durable in the blastic phase cases [22, 23].

On the 30th day of treatment, our patient developed pneumonia in a pancytopenic setting. Bosutinib is usually well-tolerated, with only mild gastrointestinal adverse events occurring [24]. Kantarjian et al. evaluated the safety of Bosutinib in 570 patients and showed 42% thrombocytopenia (30% grades 3-4), 28% anemia (14% grades 3-4), and 19% neutropenia (14% grades 3-4). Myelosuppression occurred after 22 days of initiation and lasted for a median of 14 days (range: 1–1,373 days). Myelosuppression was mostly detected in patients who received prior therapy. This myelosuppression was controlled with different treatment modifications, which included 46% temporarily stopping treatment, 32% with a dose reduction, 10% with growth factor support, and 1% with a transfusion requirement. Unfortunately, 7% of patients discontinued treatment because of severe myelosuppression [25, 26].

CNS involvement should be considered in all lymphoid blastic phase cases. Short-term responses can be achieved with intrathecal cytarabine and Mtx concurrently with radiotherapy [27]. Dasatinib has an advantage in CNS involvement, compared with imatinib, due to its SRC/BCR-ABL TK inhibition abilities and its blood-brain barrier penetration [28]. Although there are no reports that have demonstrated bosutinib's efficacy in CNS involvement, we continued with the treatment after the suspected CNS involvement was determined because he had been unresponsive to other TKIs.

The blast phase in CML is a challenging condition due to a lack of treatment options and its low long-term response rates. This is the first case in literature demonstrating the effect of bosutinib in blast phase CML as an induction therapy in an older patient. Bosutinib, as a third-generation TKI, can be an alternative agent in elderly blastic-phase CML patients with a high tolerability profile and increased survival. If bosutinib is found to be effective in CNS involvement, it should be investigated further.
